# PHF5A通过调节PI3K/AKT通路促进非小细胞肺癌的增殖和迁移

**DOI:** 10.3779/j.issn.1009-3419.2023.102.01

**Published:** 2023-01-20

**Authors:** Houhui WANG, Fanglei LIU, Chunxue BAI, Nuo XU

**Affiliations:** ^1^200032 上海，复旦大学附属中山医院呼吸科; ^1^Department of Pulmonary Medicine, Zhongshan Hospital, Fudan University, Shanghai 200032, China; ^2^276500 日照，日照市莒县人民医院; ^2^People’s Hospital of Juxian, Rizhao 276500, China

**Keywords:** PHF5A, 肺肿瘤, 增殖, 迁移, PHF5A, Lung neoplasms, Proliferation, Migration

## Abstract

**背景与目的:**

非小细胞肺癌（non-small cell lung cancer, NSCLC）的诊断及治疗仍然是目前研究的热点与难点，探索NSCLC增殖和转移的分子机制及寻找新的靶点是当前研究的焦点。PHD锌指结构域蛋白5A（plant homodomain finger-like domain-containing protein 5A, PHF5A）在维持正常细胞的基本生物学功能中起重要作用。本研究旨在探讨PHF5A在NSCLC细胞增殖、转移中的作用及分子机制。

**方法:**

采用慢病毒转染方法构建A549、PC-9 PHF5A稳定过表达细胞株；采用siRNA技术构建PHF5A抑制的H292和H1299细胞株。利用流式细胞技术检测细胞周期。采用克隆形成、MTT法、细胞增殖计数检测细胞增殖情况，采用细胞迁移实验及细胞划痕实验检测细胞体外迁移能力变化。利用A459稳定过表达细胞株构建裸鼠成瘤模型，并观察比较PHF5A过表达细胞与对照组细胞的成瘤能力。用Western blot方法分析细胞内PHF5A及PI3K/AKT通路及其下游p21、c-Myc的表达变化。

**结果:**

与对照组相比，PHF5A过表达组的PHF5A表达明显增加，PHF5A抑制组的PHF5A表达明显减少（P<0.05）；PHF5A过表达组24 h、48 h、72 h细胞增殖率均明显升高，抑制PHF5A表达组24 h、48 h、72 h细胞增殖率明显下降（P<0.05）。成瘤实验中，与对照组相比，PHF5A过表达组成瘤速度明显加快，瘤体体积明显增加（P<0.05）。利用Transwell迁移实验以及划痕实验证实PHF5A过表达组细胞的迁移能力较对照组明显增加，抑制PHF5A的表达可以降低细胞的迁移能力（P<0.05）。同时，抑制PHF5A的表达使细胞周期被抑制在G_1_期/S期，PI3K、磷酸化AKT和下游c-Myc表达明显减少（P<0.05），p21表达明显升高（P<0.05）。

**结论:**

PHF5A表达增加可以促进NSCLC细胞增殖及迁移，PI3K/AKT信号通路可能是其作用的机制之一。

## Abstract (en)

**Background and objective:**

There have been many significant advances in the diagnosis and treatment of non-small cell lung cancer (NSCLC). However, the mechanism underlying the progression of NSCLC is still not clear. Plant homodomain finger-like domain-containing protein 5A (PHF5A) plays an important role in processes of chromatin remodeling, morphological development of tissues and organs and maintenance of stem cell pluripotency. This study aims to investigate the role of PHF5A in the proliferation and migration of NSCLC.

**Methods:**

A549 and PC-9 PHF5A overexpression cell lines were constructed. PHF5A expression was decreased in H292 and H1299 cells by using siRNA. Flow cytometry was used to detect the cell cycle. MTT assay and clone formation assay were used to examine the proliferative ability of NSCLC, while migration assay and wound healing assay were performed to evaluate the ability of migration. Western blot analysis was used to measure the expressions of PI3K, p-AKT and the associated downstream factors.

**Results:**

Up-regulation of PHF5A in A549 and PC-9 cells increased the proliferation rate, while down-regulation of PHF5A in H292 and H1299 cells inhibited the proliferation rate at 24 h, 48 h and 72 h (P<0.05). The metastatic ability was elevated in the PHF5A-overexpresion groups, while reduced in the PHF5A-down-regulation group (P<0.05). In addition, reduced expression of PHF5A induced cell cycle arrest at G_1_/S phase (P<0.05). Furthermore, decreased expression of PHF5A reduced the expression levels of PI3K, phosphorylation of AKT, c-Myc (P<0.05) and elevated the expression of p21 (P<0.05).

**Conclusion:**

These results demonstrated that PHF5A may play an important role in progression of NSCLC by regulating the PI3K/AKT signaling pathway.

肺癌是世界范围内常见的恶性肿瘤，其发病率和死亡率均居前列^[1]^。非小细胞肺癌（non-small cell lung cancer, NSCLC）占肺癌的85%左右。靶向治疗以及免疫治疗的出现给NSCLC的治疗带来了革命性变化。然而，对于晚期患者的预后仍然不理想，总体5年生存率仍在15%左右^[2]^。因此，进一步研究肺癌的分子机制，寻找新的潜在的癌症治疗靶点将为NSCLC诊治提供新的思路。

PHD锌指结构域蛋白5A（plant homodomain finger-like domain-containing protein 5A, PHF5A）在细胞核中广泛表达，它编码110个氨基酸，属于一种高度保守的小转录启始因子。主要参与调节基因的转录及选择性剪接，对维持正常细胞的基本生物学功能起重要作用。PHF5A是参与可变剪接（alternative splicing, AS）的关键剪接因子，直接参与蛋白质间的相互作用或通过RNA剪接途径调控下游基因。如果将其敲除则破坏了多个必需基因的剪接，可导致细胞周期阻滞和活力丧失^[3⇓-5]^。已有研究^[6]^发现PHF5A是肺鳞癌RNA结合蛋白网络中的关键环节；并有研究^[7,8]^证明，它在大肠癌和胰腺癌的发生发展过程中起到重要作用。这些发现提示PHF5A在肿瘤的发生发展中可能作为基因的转录调控因子对细胞周期、促癌基因表达等过程发挥重要作用。但是它在肺癌尤其是NSCLC中的分子和生物学功能还不是很清楚。在本研究中，我们探讨了PHF5A在NSCLC增殖和迁移中的作用，并探讨了其发挥作用的可能机制。

## 1 材料与方法

### 1.1 细胞增殖实验

A549细胞在含10%FBS的RPMI-1640培养基中培养，经0.25%胰酶消化后，于800 rpm离心5 min，然后用完全培养基重悬细胞并计数，其中每孔接种1,000个细胞，于培养箱中分别培养5 d。在96孔板的每个孔中加入10 μL MTT，于培养箱中孵育4 h后，弃去MTT和完全培养基，然后加入100 μL DMSO，于培养箱中孵育10 min，中间去除摇晃一次，然后酶标仪（TECAN Nanoquant, Switzerland）中检测各孔在490 nm处的吸光光度值。上述培养条件均为37 ^o^C、5%CO_2_加湿培养箱。

### 1.2 细胞迁移实验

取对数生长期细胞，调整细胞密度为1×10^5^个/mL，将Transwell小室放入24孔培养板中，在小室外加入含10%FBS完全培养液或1%FBS培养液（对照组），小室内接种1×10^4^个细胞，37 ^o^C、5%CO_2_、饱和湿度培养箱中孵育6 h，PBS漂洗后，Cooomassie Blue室温染色适当时间。倒置显微镜下×10物镜计数贴附于小室多孔膜上的总细胞数（N0），棉签擦去小室多孔膜上方细胞，计数残留在小室多孔膜下方的细胞数（N1），每一张膜随机选取5个视野拍照并进行统计。按以下公式计算细胞迁移率：细胞迁移率=N1/N0×100%。

### 1.3 细胞克隆形成实验

人NSCLC细胞A549过表达PHF5A基因的稳转细胞系A549-PHF5A和相应的对照组细胞A549-vector分别经0.25%含EDTA的胰酶消化后，用含10%FBS的完全培养基终止消化，于800 rpm离心5 min，然后用完全培养基将沉淀重悬成单个细胞并计数，在12孔板的每个孔中接种含600个细胞的1 mL完全培养基，置于37 ^o^C、5%CO_2_培养箱中培养1周，将12孔板中的培养液完全吸出后，在每个孔中加入1 mL 100%甲醇，室温下固定10 min，固定完成后，用一定量的0.1%的结晶紫进行染色30 min（结晶紫染液用75%的乙醇配置）。染色结束后用水清洗残余染料并拍照，并在镜下随机选取5个视野，计数超过100个细胞的克隆形成的数量。

### 1.4 免疫印迹法

用Bradford方法测量蛋白含量。SDS-PAGE电泳分离裂解液并转移到PVDF膜上。TBST洗膜3次，封闭过的膜分别用一抗、二抗孵育。配制ECL显色液，并在暗室X线曝光显影。并用Image J分析软件测量每个特异条带的灰度值。抗体：PI3K（Cell Signaling Technology, #17366），AKT（Cell Signaling Technology, #4691），p-AKT（Cell Signaling Technology, #4060），c-Myc（Cell Signaling Technology, #18583），p21（Cell Signaling Technology, #2947）。

### 1.5 划痕实验

将H1299-NC及抑制PHF5A表达的H1299-PHF5A细胞，A549-vector和稳转过表达细胞株A549-PHF5A细胞分别采用含10%FBS的1640培养基，于37 ^o^C、5%CO_2_、pH值7.2-7.4的无菌恒温培养，待细胞长到80%融合度时分别用含EDTA的0.25%的胰酶消化，在6孔板的每孔接种细胞2×10^6^个，24 h后划线，划线后的0 h和48 h拍照。使用Image J软件打开图片后，随机划取6条-8条水平线，计算细胞间距离的均值。

### 1.6 体内成瘤实验

使用SPF级环境中喂养的4周龄-5周龄的雄性BALB/CA裸鼠（购于中国科学院上海分院）。取5×10^6^个PHF5A高表达A549细胞和对照组细胞，分别皮下注射到裸鼠右侧皮肤处，每组6只。5周后，收获瘤体并进行统计分析。

瘤体体积（V）=长×宽×宽/2

### 1.7 统计学分析

采用SPSS 17.0用于数据的统计学处理，所有数据用均数±标准差（Mean±SD）表示，组间比较采用方差分析（ANOVA）法检验。P<0.05认为差异有统计学意义。

## 2 结果

### 2.1 减少NSCLC细胞中PHF5A表达可以抑制细胞体外增殖

为了研究PHF5A在NSCLC增殖中的作用，利用siRNA技术抑制NSCLC H292和H1299中PHF5A的表达。与对照组相比，抑制组细胞中PHF5A表达明显降低（图1A）。H292-NC组细胞在24 h、48 h、72 h的增殖率分别为30.2%、47.3%、99.3%，H292-siPHF5A组细胞的增殖率分别为11.5%、18.2%、43.1%；H1299-NC组细胞在24 h、48 h、72 h的增殖率分别为47.8%、77.4%、98.6%，H1299-siPHF5A组细胞的增殖率分别为41.2%、51.4%、60.8%。上述结果说明PHF5A抑制组的各个时间的细胞增殖率均比相对应的对照组的增殖率明显减少（P<0.05，图1B）。因此下调PHF5A的表达，可以明显抑制NSCLC H292和H1299细胞的增殖。同时，流式细胞检测结果显示，抑制PHF5A的表达可以使更多的细胞停留在G_1_期/S期（图1C、图1D）。

**图1:**
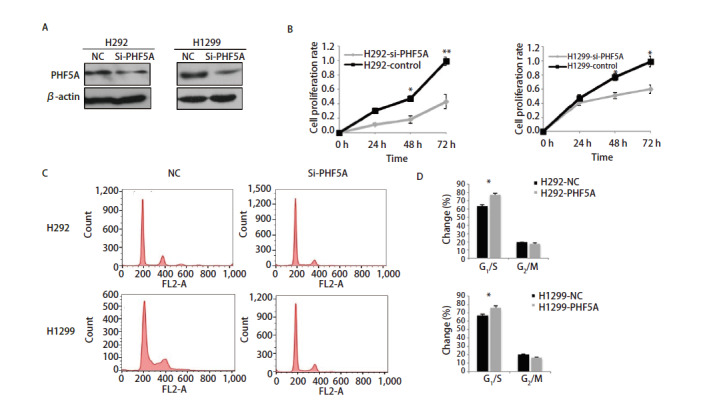
减少NSCLC细胞中PHF5A表达降低细胞体外增殖。A：siRNA技术抑制NSCLC H292和H1299中PHF5A的表达，Western blot法检测各组细胞中PHF5A的表达水平；B：减少PHF5A表达后各组H292和H1299的细胞增殖率明显降低；C：利用流式细胞学技术检测细胞周期变化；D：细胞周期定量分析。与NC组比较，抑制PHF5A的表达可以使细胞停留在G_1_期/S期。*P<0.05，**P<0.01。

### 2.2 增加NSCLC细胞中PHF5A表达可以促进细胞体外增殖

利用A549和PC-9细胞构建PHF5A过表达细胞株，PHF5A在过表达组中的表达比对照组明显增加（图2A）。与对照组相比，A549和PC-9过表达组细胞克隆形成能力明显增加（图2B、图2C），利用MTT法检测细胞增殖率，PC-9-vector组细胞24 h、48 h、72 h的增殖率分别为41.1%、64.4%、72.8%，PC-9-PHF5A组细胞的增殖率分别为61.4%、94.1%、115.0%；A549-vector组细胞24 h、48 h、72 h的增殖率分别为14.0%、20.7%、40.1%，A549-PHF5A组细胞的增殖率分别为21.1%、28.1%、58.6%。上述结果提示PHF5A过表达组的各个时间的细胞增殖率均比相对应的对照组的增殖率明显增加（P<0.05，图2D）。因此，PHF5A表达增加可以促进NSCLC细胞A549和PC-9细胞的增殖。

**图2:**
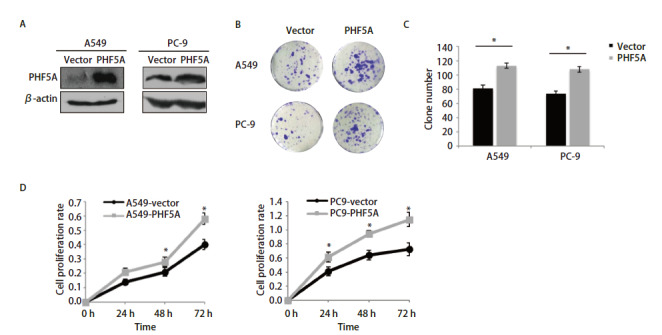
增加NSCLC细胞中PHF5A表达促进细胞体外增殖和克隆形成能力。A：构建NSCLC A549、PC-9 PHF5A稳定高表达细胞株，Western blot法检测各组细胞中PHF5A的表达水平；B：利用克隆形成实验，比较PHF5A稳定高表达和对照组细胞克隆形成能力（结晶紫染色）；C：染色后集落计数的定量分析。结果提示与对照组相比，A549和PC-9过表达组细胞克隆形成能力明显增加；D：利用MTT法检测细胞增殖率，与对照组相比，增加PHF5A表达后各组A549和PC-9的细胞增殖率明显升高。*P<0.05。

### 2.3 增加NSCLC细胞中PHF5A表达可以促进NSCLC细胞体外迁移

为了更好地研究PHF5A在NSCLC中的作用，我们利用A549和PC-9过表达细胞株进行了Transwell迁移检测。A549-PHF5A组和PC-9-PHF5A组的细胞数分别为827.7±20.5和663.0±33.4，明显多于相应对照组（A549-vector: 342.3±26.3; PC-9-vector: 239.3±25.8）（P<0.05）。结果说明增加PHF5A的表达，可以显著促进A549和PC-9细胞的迁移（图3）。同时，我们利用A549过表达细胞株以及H1299低表达细胞株分别进行了划痕实验，并在48 h后比较两组细胞与对照组之间的划痕修复能力是否具有差异。结果提示与对照组相比，A549过表达组细胞迁移速度明显增加，而H1299低表达组细胞的迁移速度明显下降（P<0.05）（图4），说明PHF5A可以显著促进NSCLC细胞的迁移能力。

**图3:**
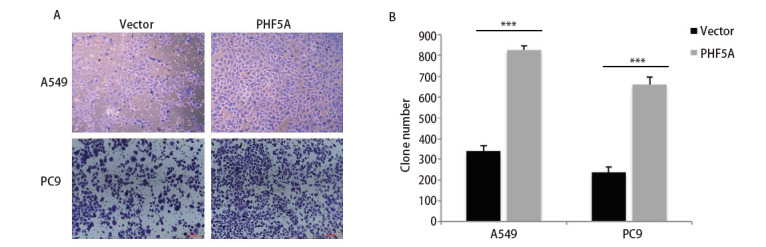
增加NSCLC细胞中PHF5A表达可以增加NSCLC细胞迁移的能力。A：利用细胞迁移实验，比较A549和PC-9过表达细胞株和对照组细胞迁移的能力（Cooomassie Blue染色，×100）；B：染色后细胞计数的定量分析。结果提示增加PHF5A的表达，可以显著促进A549和PC-9细胞的迁移能力。***P<0.001。

**图4:**
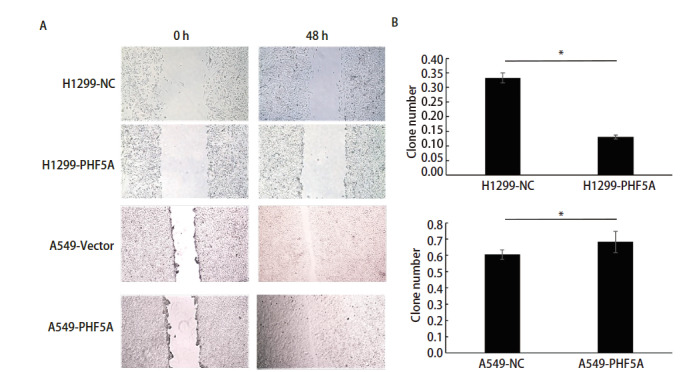
NSCLC细胞中PHF5A表达变化可以影响NSCLC细胞迁移的速度。A：利用细胞划痕实验，比较A549过表达细胞组、H1299低表达细胞组和对照组细胞在0 h、48 h的划痕愈合状态（×100）；B：迁移细胞计数的定量分析。结果提示PHF5A的表达变化可以影响NSCLC细胞的迁移能力。*P<0.05。

### 2.4 增加NSCLC细胞中PHF5A表达可以促进肿瘤细胞体内增殖

利用A549-PHF5A过表达细胞株进行体内成瘤实验，结果发现PHF5A过表达组成瘤体积[A549-PHF5A: (0.98±0.39) mm^3^]明显大于对照组[A549-vector: (0.42±0.21) mm^3^]（P<0.05），同时成瘤速度也明显增加（图5）。因此，PHF5A过表达可以促进NSCLC A549细胞体内增殖。

**图5:**
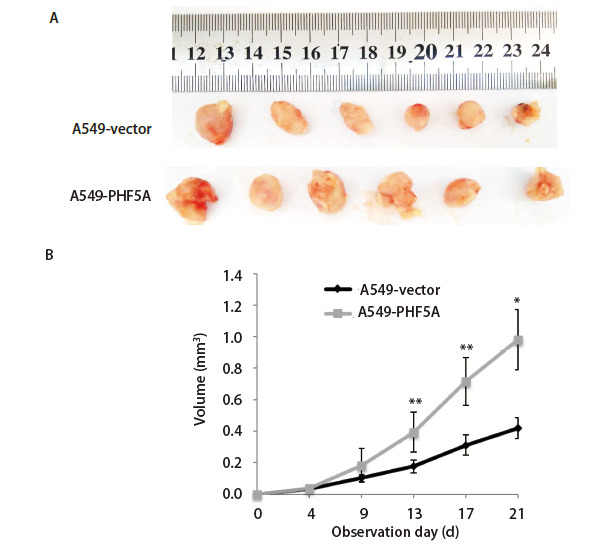
增加NSCLC细胞A549细胞中PHF5A表达可以促进在体肿瘤细胞的增长。A：切除下来的小鼠肿瘤；B：特定时间肿瘤体积。与对照组比较，过表达组瘤体体积明显增加，增殖速度加快。*P<0.05，**P<0.01。

### 2.5 PHF5A可以通过激活PI3K/AKT信号通路调节细胞增殖和转移

为了进一步探讨PHF5A促进NSCLC增殖的机制，前期我们利用基因芯片对下游通路进行了分析，发现PHF5A可能调节PI3K/AKT信号通路。因此，我们利用siRNA技术抑制了A549细胞中PHF5A的表达，并检测其对PI3K/AKT信号通路下游相关蛋白表达的影响。结果提示，与对照组相比，PHF5A表达下调后，PI3K、磷酸化AKT和下游c-Myc表达明显减少（P<0.05）；而p21表达明显升高（P<0.05）（图6）。说明PHF5A可能通过激活PI3K/AKT通路调节NSCLC细胞的增殖和迁移。

**图6:**
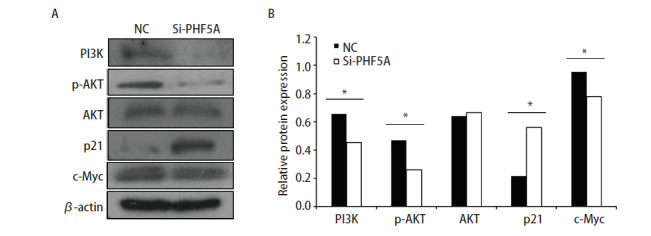
抑制NSCLC细胞A549细胞中PHF5A表达对PI3K/AKT信号通路中相关蛋白表达的影响。A：利用siRNA技术抑制了A549细胞中PHF5A的表达，Western blot法检测A549抑制组与对照组细胞中PI3K、p-AKT、AKT、p21及c-Myc的表达水平；B：半定量分析Western blot检测结果，提示PHF5A降低可以抑制PI3K、p-AKT及c-Myc的表达，而p21表达明显升高。*P<0.05.

## 3 讨论

PHF5A是高度保守的小转录启始因子，参与细胞周期的调控、细胞生长和分化^[7,9]^，对染色质重塑、组织和器官形态发展、肿瘤干细胞样表型的维持起着重要的作用^[4,9⇓-11]^。有研究^[12]^报道抑制PHF5A的表达可以抑制胶质母细胞瘤细胞的增殖，同时其在胶质母细胞瘤中的外显子识别、维持细胞扩增以及活力中发挥重要作用。另一项NSCLC的研究^[4]^中发现，PHF5A在肺癌细胞中表达升高，并且可能通过选择性剪接发挥促癌作用。PHF5A还被证实在胰腺癌细胞中可以与PAF1和DDX3相互作用，通过抑制PAF1-PHF5A-DDX3复合物，可以抑制原位胰腺癌的增殖，以及肿瘤在小鼠体内的发展。研究^[6⇓-8]^发现在结肠癌中，PHF5A乙酰化可以通过抗压力途径增进肿瘤细胞对抗应激的能力，最终促进结肠癌细胞的进展。但PHF5A在肺癌发生发展中的具体作用尚未完全明确。深入探究PHF5A在肺癌进展中的作用，有助于寻找新的潜在的癌症治疗靶点，为NSCLC诊治提供新的思路。

有研究^[6]^发现，与正常组织相比，PHF5A在NSCLC中显著上调，且PHF5A上调与患者的总生存期呈负相关。本研究通过克隆形成、MTT法、Transwell、划痕实验等方法检测了增加或抑制PHF5A的表达后NSCLC细胞的增殖和迁移能力的变化。结果显示，下调NSCLC细胞中PHF5A的表达可以抑制NSCLC细胞的增殖和迁移，升高PHF5A的表达可以促进NSCLC细胞的增殖和迁移。PHF5A的抑制可以使细胞周期停留在G_1_期/S期，进一步证实了PHF5A在NSCLC增殖中的促进作用。PI3K/AKT信号通路在肿瘤发生发展的作用已为人们所熟知，已有报道，PI3K/AKT在NSCLC、前列腺腺癌和肠癌中持续活化以及表达量增高。它可以调节肿瘤细胞的增殖和存活，其活性异常不仅能导致细胞恶化，且与肿瘤的侵袭转移行为密切相关。本研究抑制NSCLC细胞A549中PHF5A蛋白表达，并应用Western blot方法检测的PI3K/AKT通路及其下游p21、c-Myc蛋白表达的变化，初步探索了PHF5A对肺癌细胞生物学功能产生的影响可能是通过激活PI3K/AKT通路产生的。

综上所述，NSCLC细胞中PHF5A的表达增高，并可以通过影响PI3K/AKT信号通路促进肺癌细胞的增殖和迁移。而研发PHF5A的相关抑制剂，可能为预防和治疗NSCLC提供新的思路。

## References

[1] Sung H, Ferlay J, Siegel RL, et al. Global cancer statistics 2020: GLOBOCAN estimates of incidence and mortality worldwide for 36 cancers in 185 countries. CA Cancer J Clin, 2021, 71(3): 209-249. doi: 10.3322/caac.21660 33538338

[2] Tan AC, Tan DSW. Targeted therapies for lung cancer patients with oncogenic driver molecular alterations. J Clin Oncol, 2022, 40(6): 611-625. doi: 10.1200/JCO.21.01626 34985916

[3] Chang Y, Zhao Y, Wang L, et al. PHF5A promotes colorectal cancer progression by alternative splicing of TEAD2. Mol Ther Nucleic Acids, 2021, 26: 1215-1227. doi: 10.1016/j.omtn.2021.10.025 34853721 PMC8605294

[4] Yang Y, Li M, Zhou X, et al. PHF5A contributes to the maintenance of the cancer stem-like phenotype in non-small cell lung cancer by regulating histone deacetylase 8. Ann Clin Lab Sci, 2022, 52(3): 439-451. 35777798

[5] Teng T, Tsai JH, Puyang X, et al. Splicing modulators act at the branch point adenosine binding pocket defined by the PHF5A-SF3b complex. Nat Commun, 2017, 8: 15522. doi: 10.1038/ncomms15522 28541300 PMC5458519

[6] Zhao S, Liu Q, Li J, et al. Construction and validation of prognostic regulation network based on rna-binding protein genes in lung squamous cell carcinoma. DNA Cell Biol, 2021, 40(12): 1563-1583. doi: 10.1089/dna.2021.0145 34931870

[7] Wang Z, Yang X, Liu C, et al. Acetylation of PHF5A modulates stress responses and colorectal carcinogenesis through alternative splicing-mediated upregulation of KDM3A. Mol Cell, 2019, 74(6): 1250-1263.e6. doi: 10.1016/j.molcel.2019.04.009 31054974

[8] Karmakar S, Rauth S, Nallasamy P, et al. RNA polymerase II-associated factor 1 regulates stem cell features of pancreatic cancer cells, independently of the PAF1 complex, via interactions with PHF5A and DDX3. Gastroenterology, 2020, 159(5): 1898-1915.e6. doi: 10.1053/j.gastro.2020.07.053 32781084 PMC7680365

[9] Begum NA, Haque F, Stanlie A, et al. Phf5a regulates DNA repair in class switch recombination via p400 and histone H2A variant deposition. EMBO J, 2021, 40(12): e106393. doi: 10.15252/embj.2020106393 33938017 PMC8204862

[10] Yu AQ, Wang J, Jiang ST, et al. The SIRT7-induced PHF5A decrotonylation regulates aging progress through alternative splicing-mediated downregulation of CDK2. Front Cell Dev Biol, 2021, 9: 710479. doi: 10.3389/fcell.2021.710479 34604215 PMC8484718

[11] Cretu C, Gee P, Liu X, et al. Structural basis of intron selection by U2 snRNP in the presence of covalent inhibitors. Nat Commun, 2021, 12(1): 4491. doi: 10.1038/s41467-021-24741-1 34301950 PMC8302644

[12] Hubert CG, Bradley KR, Ding Y, et al. Genome-wide RNAi screens in human brain tumor isolates reveal a novel viability requirement for PHF5A. Genes Dev, 2013, 27: 1032-1045. doi: 10.1101/gad.212548.112 23651857 PMC3656321

